# The Effect of Maternal Antenatal Care Utilisation on Childhood Acute Respiratory Infection: A Systematic Review and Meta-Analysis

**DOI:** 10.3390/ijerph22111627

**Published:** 2025-10-26

**Authors:** Melash Asresie, Yibeltal Bekele, Don Vicendese, Mehak Batra, Bircan Erbas

**Affiliations:** 1 School of Psychology and Public Health, La Trobe University, Melbourne, VIC 3083, Australia; m.asresie@latrobe.edu.au (M.A.); y.bekele@latrobe.edu.au (Y.B.); b.erbas@latrobe.edu.au (B.E.); 2 School of Public Health, Bahir Dar University, Bahir Dar P.O. Box. 79, Ethiopia; 3 School of Population and Global Health, The University of Melbourne, Melbourne, VIC 3010, Australia; don.vicendese@unimelb.edu.au; 4 School of Computing, Engineering and Mathematics Science, La Trobe University, Melbourne, VIC 3083, Australia

**Keywords:** pneumonia, antenatal care, acute respiratory infection, under-five children, systematic review

## Abstract

Background: Antenatal care (ANC) offers a valuable opportunity to reduce the risk of acute respiratory infections (ARIs) in children under five; however, its impact remains less understood and has not been comprehensively synthesised. This systematic review aimed to assess evidence on the association between ANC utilisation and ARIs in children under five. Methods: A systematic search was conducted in PubMed, CINAHL, Scopus, Web of Science, and Google Scholar for studies published between 2000 and 2025. PRISMA 2020 guidelines were followed in reporting. A qualitative synthesis was performed for all ARI outcomes, and a meta-analysis was conducted for pneumonia. Results: Eleven observational studies assessed the association between ANC utilisation and ARIs. Three ARI-related outcomes were identified: pneumonia (*n* = 4), pertussis (*n* = 2), and general ARIs (*n* = 5). ANC attendance was generally associated with reduced odds of respiratory infections across studies. The pooled analysis of two case–control studies (*n* = 2; total *n* = 956) showed a non-significant association between ANC attendance and pneumonia in children (pooled OR = 1.46; 95% CI: 0.91, 2.35; I^2^ = 0%). Conclusion: Our review suggests a potential protective effect of ANC, though evidence from the pneumonia-focused meta-analysis was inconclusive. Improving access to quality ANC may reduce respiratory infections, but more studies are needed across different populations.

## 1. Introduction

Maternal and child health remains a global public health priority, particularly in low- and middle-income countries where preventable diseases continue to contribute significantly to under-five mortality [1]. Among these, acute respiratory infections (ARIs) remain the leading cause of mortality in young children [2]. Despite a 59% decline in child mortality over the past three decades, 4.8 million children under five still die annually, mostly from preventable causes [3]. ARIs, particularly pneumonia, remain a major contributor, responsible for 700,000 deaths each year, with nearly 70% occurring in Africa and South Asia [4]. ARIs also drive high rates of paediatric hospitalisations, accounting for up to 61% of illness-related visits to emergency and 30% of admissions [5,6,7]. Furthermore, ARIs have long-term consequences over the life course, including chronic respiratory conditions and premature mortality in adulthood [8,9].

Eliminating preventable ARI deaths by 2030 is a global health priority, with the Sustainable Development Goals targeting a reduction in under-five mortality to 25 per 1000 live births [1]. More than 84% of infection-related child deaths could be prevented through improved coverage of the maternal and newborn continuum of care and quality of interventions such as Antenatal care (ANC), skilled birth attendance, postnatal care, newborn care, and childhood immunisation [10]. However, millions of children, particularly in low- and middle-income countries (LMICs), lack access to preventive care. In 2023, 21 million children remain unvaccinated or under-vaccinated, with over half from seven LMICs (Nigeria, India, Ethiopia, Democratic Republic of Congo, Sudan, Indonesia, and Yemen) [11]. Gaps in maternal and infant care further exacerbate risks, particularly due to inadequate care-seeking behaviours and limited awareness of illness in offspring during early life [12,13].

ANC could potentially reduce the risk of ARIs by improving maternal and child health through disease screening and promoting healthy behaviours. It could support the early detection and management of maternal conditions, such as infections, anaemia, and foetal growth restriction, which can reduce the risk of disease transmission to the newborn and strengthen neonatal immunity [14].

ANC also promotes maternal immunisation and nutritional supplementation, which can reduce low birth weight and protect mothers and babies from diseases. It also provides education on postnatal care, including exclusive breastfeeding and infant immunisations [15,16]. Moreover, it plays a key role in ensuring that mothers continue with postnatal and newborn healthcare, reducing the likelihood of infections and contributing to increased child survival [17,18]. Despite all pregnant women being advised to attend a minimum of eight ANC visits, with the first visit ideally taking place during the first trimester of pregnancy, utilisation and adherence to the recommended visits remain poor, particularly in LMICs. Studies show that 11.2% of women in LMICs never attended ANC, a staggering 50.1% initiated it late, and only 11.3% completed the recommended eight visits [19]. In Africa, just 37.2% of mothers attend ANC in the first trimester [20], and only 9% had eight or more ANC visits [21], contributing to poor maternal and child health outcomes.

While ANC’s role in improving child health and reducing the risk factors is well-documented [22,23,24], evidence of its potential long-term effects on ARIs remains limited. To our knowledge, no systematic review and meta-analysis has comprehensively examined the relationship between ANC and childhood ARIs. Given the significant burden of ARIs, particularly in LMICs, exploring this association is crucial for achieving the global goal of ending preventable pneumonia deaths and reducing overall child mortality. Insights gained could inform strategies to support women during and after childbirth, potentially improving child health outcomes. This review aims to synthesise existing evidence on the impact of ANC on childhood ARIs, thereby addressing a critical knowledge gap and guiding future research and targeted public health interventions in LMICs.

## 2. Methods

This systematic review was conducted following the Preferred Reporting Items for Systematic Reviews and Meta-Analyses (PRISMA) reporting guidelines (Table S1) [25].

### 2.1. Eligibility Criteria

#### 2.1.1. Population

Children under five years of age (0–59 months) were included.

#### 2.1.2. Exposure

The primary exposure was ANC use, defined as any visit to a health facility that is used for ANC during pregnancy.

#### 2.1.3. Outcome

Any ARIs, including pneumonia, pertussis, and upper or lower respiratory tract infection, as defined by the authors.

#### 2.1.4. Study Design

All observational studies (cross-sectional, case–control, and cohort) assessing the association between ANC use and ARI among children under five were included. Whereas case reports, case series reports, grey literature, and non-primary research (e.g., narrative reviews, editorials, commentaries) were excluded. Additionally, studies that did not assess ARI as a dependent variable/outcome and ANC use as an exposure or factor variable were excluded.

#### 2.1.5. Language and Time Frame

The review included studies published in English from 2000 onward. The year 2000 was chosen to align with increased global attention to maternal and child health following the Millennium Development Goals [26].

### 2.2. Search Strategy

A comprehensive literature search was conducted using Medline, CINAHL, Scopus, and Web of Science. The initial search was conducted on 17 January 2025, and the last search on 2 February 2025. Google Scholar supplemented database searches, with the first 10 pages of results screened. To ensure thoroughness, reference lists of all included studies were reviewed for additional relevant studies. The search strategy was developed in consultation with a research librarian and included Medical Subject Headings (MeSH) terms, keywords, and Boolean operators (Table S2). The search terms combined keywords related to the population (e.g., “under-five children”, “infants”, “preschool”), the exposure (e.g., “antenatal care”, “prenatal check”), and the outcome (e.g., “acute respiratory infection”, “pneumonia”).

### 2.3. Study Selection and Data Extraction

All search results were imported into EndNote 20.6, and duplicates were automatically removed using Covidence. Two reviewers (M.A. & Y.B.) independently screened the title and abstract. If the articles met the inclusion criteria, a full-text review was performed. Any disagreements were resolved through discussion with senior authors (M.B. & B.E.). A standardised Microsoft Excel data extraction form was used to collect relevant details from each included study. The extracted information included study characteristics such as author, publication year, study country, and country income level. Additionally, methodological details were recorded, including study design, sample size, and measurement methods for ANC and ARI. Finally, the key findings for each study were documented to facilitate a comprehensive synthesis of the results.

### 2.4. Quality Assessment

The Newcastle-Ottawa scale (NOS) was used to assess the risk of bias in the included studies [27], evaluating them across three domains: selection, comparability, and outcome assessment. Based on the total NOS score, studies were classified as having a lower risk of bias (≥7 points), moderate risk of bias (5–6 points), or high risk of bias (≤4 points).

### 2.5. Data Synthesis and Analysis

Both qualitative synthesis and meta-analysis were employed. A qualitative synthesis was conducted following the Synthesis Without Meta-analysis (SWiM) guidelines [28]. Studies were categorised by outcome (Pneumonia, ARI, and pertussis), and their findings were summarised thematically. We also conducted a meta-analysis using Stata software version 17 to synthesise eligible studies. A random-effects model with a 95% confidence interval (CI) was used to estimate the pooled effect size of ANC use on childhood ARI. A forest plot was used to summarise the findings of the pooled effects, and heterogeneity across the studies was assessed using I^2^ tests.

## 3. Results

### 3.1. Search Results and Characteristics of the Studies

**Figure 1:**
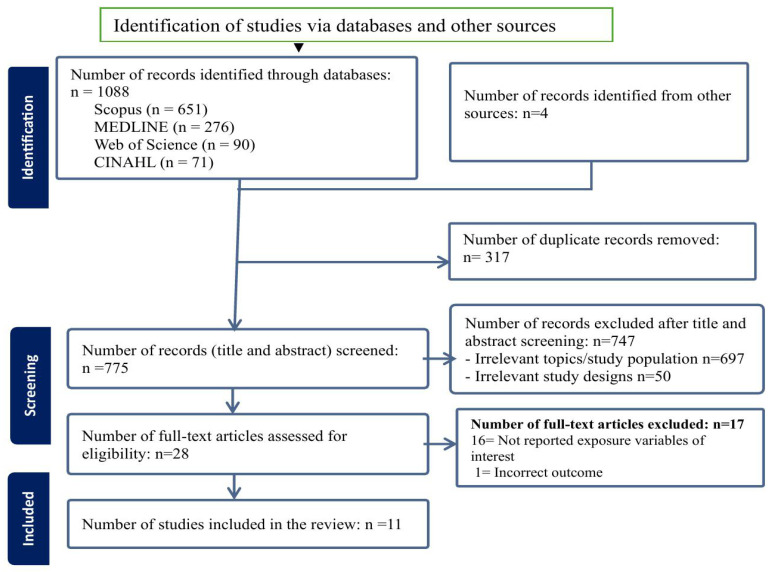
PRISMA flow diagram of the screening and inclusion process.

After screening 775 titles and abstracts, a total of 28 studies were selected for full-text review, of which 11 studies met the inclusion criteria and were included in this review (Figure 1).

The sample size of the included studies ranged from 100 neonates (<28 days) admitted to a hospital in Bangladesh [29] to 994,244 mother-infant pairs of the birth cohort in the United States (US) [30]. Geographically, eight studies were conducted in LMICs, including three studies from Ethiopia [31,32,33] and one each from China [34], Bangladesh [29], Ghana [35], Rwanda [36], and a pooled analysis of Demographic and Health Survey (DHS) data from 25 sub-Saharan African (SSA) countries was conducted using multilevel modelling. [37] The remaining three studies were conducted in high-income countries (HICs), with two in the US [30,38] and one in England [39]. In terms of study design, four studies were case–control studies [29,30,32,33], while the remaining seven were cross-sectional studies [31,34,35,36,37,38,39] (Table 1).

### 3.2. Quality Assessment of Included Studies

A thorough quality assessment of the included studies was conducted using NOS, focusing on selection, comparability, and outcome assessment domains (Table 1). Seven studies were identified as having a high risk of bias due to inadequate control of potential confounders such as residence, maternal age, and education, and insufficient detail regarding outcome measurements [29,30,31,32,33,34,39]. Four studies were rated as having a moderate risk of bias because they omitted one or more potential confounders and lacked information on participants’ response rates [35,36,37,38]. Notably, only one study [35] adequately controlled for all identified confounders in its final model. Additionally, six studies did not report participants’ non-response rates [29,31,34,35,36,37], raising concerns about potential selection bias. Several studies lacked clear descriptions of the diagnostic criteria used to define pneumonia or acute respiratory infection cases. One study mentioned that the Integrated Management of Neonatal and Childhood Illness (IMNCI) guideline was used to diagnose Pneumonia, but no specific criteria were stated [32], and two studies relied on DHS data without providing details on the measurement method [31,35]. Furthermore, the adequacy of control groups in case–control studies was often inadequately described [30,32], and three studies employed regression analysis without reporting adjusted odds ratios for non-significant variables [30,33,39] (Table S3).

### 3.3. Exposure Variable

ANC visits were measured using various approaches. Three studies treated ANC visits as discrete variables; they quantified the exact number of visits attended during pregnancy [35,38,39]. Two studies categorised ANC attendance as a binary variable (“yes” or “no”), indicating whether participants had at least one visit [32,33]. Johnson et al.’s [36] study in Rwanda differentiated between skilled and unskilled antenatal care. Other studies employed different categorical classifications, such as fewer than three versus three or more visits [29], fewer than four versus four or more visits [37], adequate or inadequate ANC (adequacy was not defined) [34], and specific ranges like 0–5, 6–12, 13–18, or 19 or more visits [30] and no visits, fewer than five visits, or five or more visits [31]. One study also considered the timing of the first ANC visit, distinguishing between those occurring before or after four months of gestational age [32].

### 3.4. Outcome Variables

Of the total 11 included studies, four focused on pneumonia [29,32,33,34], two on pertussis [30,38], and five on ARI [31,35,36,37,39]. Two pneumonia studies were conducted in neonates [29,34], while the remaining two focused on children under two and five years old [32,33]. Similarly, four of the five ARI studies examined children under five years [31,35,36,37], while one focused on children under 2 years of age [39]. A study conducted in England on respiratory infections reported outcomes in three different healthcare utilisation metrics: primary healthcare consultations (PHC), accident and emergency (A&E) attendance, and hospital admissions (HA), at least one with respiratory infection for each metric over the two years [39].

Both pertussis studies [30,38] were conducted in infants, with one study further distinguishing cases based on the 8-week and 12-week age groups [38]. Although both studies used data from the 2013–2014 California birth cohort, they targeted different populations. One study exclusively included infants born to mothers who received the tetanus, diphtheria, and acellular pertussis (TdaP) vaccine during the prenatal and postnatal periods [38], whereas the other study [30] examined all infants born in the same year, regardless of maternal vaccination status (Table 2).

### 3.5. Findings of Included Studies

#### 3.5.1. Pneumonia

Among the four studies conducted in LMIC examining the association between ANC and childhood pneumonia, two reported statistically significant associations [29,34]. Choudhury et al. [29] found that neonates whose mothers attended fewer than three ANC visits had significantly higher odds of developing pneumonia than those whose mothers attended three or more ANC visits (aOR = 168.90, 95% CI: 8.0, 3559.20). This study utilised physical examinations and chest X-rays to diagnose pneumonia. Similarly, Yang et al. [34] reported that inadequate ANC was associated with increased odds of neonatal pneumonia (aOR = 24.90, 95% CI: 21.20, 28.60), with diagnoses confirmed through blood culture tests. In Choudhury et al.’s [29] study, 45% of mothers received adequate ANC, defined as three or more visits during pregnancy. In contrast, Yang et al. [34] reported that 70% of mothers received adequate ANC, although the study did not specify the criteria used to define adequacy. In two Ethiopian studies, approximately 90% of mothers reported attending at least one ANC visit during pregnancy: 90% in Yadate et al. [33] and 89% in Workineh et al. [32]. Despite this high attendance, neither study found a statistically significant association between ANC and childhood pneumonia when employing regression analyses after adjusting for adequate confounders. Yadate et al. [33] observed that children aged 2–59 months whose mothers had at least one ANC visit exhibited 43% higher odds of developing pneumonia compared to those whose mothers had no ANC visits (cOR = 1.43, 95% CI: 0.84, 2.24). The study utilised multiple regression analysis, adjusted for maternal age and residence; however, it did not report results for variables that were not statistically significant. Similarly, Workineh et al. [32] found that children under two years of age whose mothers attended at least one ANC visit had higher odds of developing pneumonia compared to those whose mothers had no visits (aOR = 1.97, 95% CI: 0.34, 11.40). The study also noted a potential protective effect when the first ANC visit occurred after four months of gestation (aOR = 0.67, 95% CI: 0.41, 1.07). Importantly, this study did not adjust for other important variables such as maternal age and place of residence (Table 2).

#### 3.5.2. Pertussis

Two studies conducted in the US [30,38] investigated the relationship between ANC visits and infant pertussis, with both reporting no significant association. The 2016 cross-sectional study found that each additional ANC visit, treated as a continuous measure variable, was associated with 1.05 times higher odds of contracting pertussis at 8 weeks (aOR = 1.05, 95% CI: 0.97, 1.13) and 1.02 times higher odds at 12 weeks (aOR = 1.02, 95% CI: 0.97, 1.10) [38]. In this study, 58% of women received the Tdap vaccine during pregnancy. Their mean number of ANC visits was 12.4 (IQR: 10–14). The remaining 42% of women received Tdap within two weeks postpartum, with a mean ANC visit count of 11.8 (IQR: 10, 14). The 2018 case–control study similarly reported no significant association between ANC visits and pertussis in their unadjusted analysis [30]. Specifically, the odds of pertussis among infants whose mothers attended five or fewer ANC visits was 1.3 (cOR = 1.3, 95% CI: 0.9, 1.8), 0.9 for six to twelve visits (cOR = 0.9, 95% CI: 0.8, 1.1), 1.0 for 13–18 visits (cOR = 1.0, 95% CI: 0.9, 1.2), and 1.1 for 19 or more visits (cOR = 1.1, 95% CI: 0.5, 1.5). The study also noted that 4% of women received five or fewer ANC visits during pregnancy, while 60.0%, 31.5%, and 4.5% received 6–12, 13–18, and 19 or more visits, respectively (Table 2).

#### 3.5.3. Acute Respiratory Infection

Among four LMIC studies (Africa) and one HIC study (England) [31,35,36,37,39] examining the relationship between ANC visits and ARI, only two studies found statistically significant associations [36,39]. Johnson et al. [36] found that children under five whose mothers received ANC visits from skilled professionals had 22% lower odds of developing ARI compared to those born to mothers who received ANC from unskilled personnel (aOR = 0.78, 95% CI: 0.61, 0.99); however, the study did not report the frequency of ANC visits. Similarly, Buchana et al. [39] reported that each additional ANC visit, treated as a continuous measure variable, was associated with a 4% reduction in the odds of A&E visits for respiratory infection in children under two years old (aOR = 0.96, 95% CI: 0.92, 0.99). Additionally, this study examined the potential effect of ANC visits on primary health care (PHC) consultations and hospital admissions (HA) due to respiratory infection. ANC was not found to be significantly associated with either PHC (cOR = 1.02, 95%CI: 0.98,1.06) or HA (cOR = 1.00, 95%CI: 0.96, 104). The study employed multiple regression; however, results for non-statistically significant variables were not reported. The remaining three studies did not find a statistically significant association between ANC visits and ARI when regression techniques were applied to model association [31,35,37]. Turkson & Ahiabo [35] observed a 0.6% decrease in the likelihood of ARI with each additional ANC visit, using a continuous measure of ANC (marginal effect = −0.006, 95% CI not reported). Bokoro et al. [31] reported that children whose mothers had no ANC visits, or fewer than five visits, had 1.15 (aOR = 1.15, 95% CI not reported) and 1.20 (aOR = 1.20, 95% CI not reported) times higher odds of developing ARI under five years of age compared to those who received five or more ANC visits. Similarly, Ahmed et al. [37] using data from 25 SSA countries, found that children whose mothers had fewer than four ANC visits had 4% lower odds of contracting ARI than those whose mothers received four or more ANC visits (aOR = 0.96, 95% CI: 0.90, 1.02). In the Turkson & Ahiabor [35] study, mothers received an average of 6.8 ANC visits (SD of 6.53). Similarly, Ahmed et al. [37] found that 56.8% of mothers received four or more ANC visits, while Bokoro et al. [31] reported that 20.9% of mothers received five or more ANC visits, and 33.7% did not receive any ANC services (Table 2).

### 3.6. Meta-Analysis

**Figure 2:**
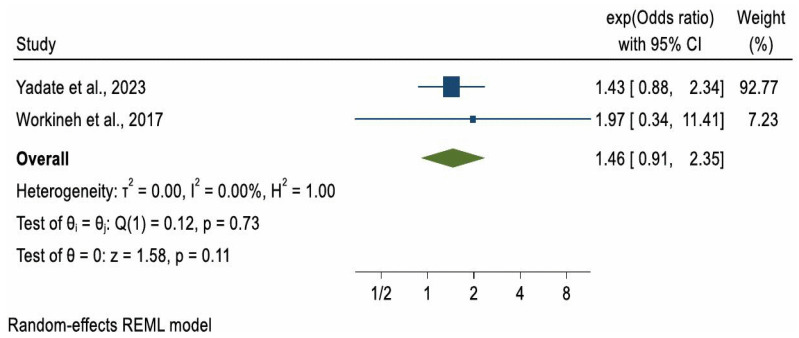
Forest plot of 2 studies [32,33] on the effect of ANC use on childhood Pneumonia.

Due to substantial heterogeneity in study methodologies, particularly in how ANC was measured, a meta-analysis was not conducted for ARI and pertussis outcomes. However, a meta-analysis could be considered for pneumonia. Of the four studies reporting on pneumonia, two case–control studies with a combined sample of 956 participants [32,33] met the criteria for meta-analysis. The pooled odds ratio (OR) for pneumonia among children whose mothers had at least one ANC visit compared to those with no ANC visits was 1.46 (OR = 1.46, 95% CI: 0.91, 2.35), indicating higher but non-significant odds of pneumonia in the ANC-exposed group (*p* = 0.11). A formal test for publication bias was not conducted due to the small number of included studies, as such tests are unreliable when fewer than ten studies are available. Between-study heterogeneity was low (I^2^ = 0.00%) (Figure 2).

## 4. Discussion

To our knowledge, this is the first systematic review to synthesise evidence from 11 observational studies examining the association between ANC utilisation and ARIs in children under five. While the findings suggest a potential protective effect of ANC in reducing childhood respiratory morbidity, the wide variation in how ANC was measured across studies highlights the need for standardised definitions and consistent reporting to enable comparability and strengthen maternal and child health research.

In the two studies where ANC showed no statistically significant effect, ANC was minimally defined as at least one visit versus none [32,33]. A meta-analysis of these studies, which used this minimal definition, also found no significant association between ANC and childhood pneumonia (pooled OR = 1.46; 95% CI: 0.91–2.35). However, this finding may reflect limited statistical power due to the small combined sample size (*n* = 956 across two studies), rather than the absence of a true effect. In contrast, studies where ANC was defined as adequate or inadequate [29,34] reported significantly lower odds of pneumonia. This supports the importance of completing the recommended schedule of visits, which provides access to maternal immunisation, nutritional supplementation, and health education. WHO recommends eight ANC contacts for optimal maternal and newborn outcomes [40], and other studies have shown that more visits are linked with more comprehensive care, including counselling on child health [41]. Environmental conditions may also explain discrepancies. For example, Yadate et al. [33] reported that 87% of households lacked or used traditional latrines, increasing exposure to pathogens and risk of diarrhoea [42]. Combined with high rates of child malnutrition (e.g., stunting and wasting), such factors weaken immunity and heighten susceptibility to pneumonia [43], potentially masking ANC’s more modest indirect effects. Diagnostic differences may also contribute: Choudhury et al. [29] and Yang et al. [33] used chest X-rays and blood cultures, whereas Workineh et al. [32] and Yadate et al. [33] relied on IMNCI guidelines, which may lack specificity and introduce potential misclassification bias. Finally, the child’s age may be relevant. The studies showing ANC benefits [29,34] were in neonates, who may be protected by maternal antibodies, exclusive breastfeeding, and reduced exposure to pathogens. In contrast, studies finding no effect [32,33] included older children, exposure to an increasingly environment and may begin consuming solid foods, thereby increasing infection risk [44].

Another key finding of this review is that five studies assessed the association between ANC and ARI, but only two studies reported a statistically significant reduction in ARI odds among children whose mothers attended ANC [36,39]. The remaining three studies, conducted in Ethiopia, Ghana, and 25 SSA countries, found no notable impact [31,35,37]. A common feature of these studies was the high proportion of rural households (57–81%), where access to healthcare and preventive services is often limited [45,46]. For example, Bokoro et al. [31] found ARI prevalence was substantially higher in rural (13.1%) than urban (2.7%) settings, highlighting the disproportionate burden of infection. Moreover, environmental hazards such as reliance on biomass fuels for cooking were nearly universal. Ahmed et al. [37] reported that 87% of mothers used polluting fuels, contributing to indoor air pollution and increased risk of respiratory illness in children [47]. This underscores the need to interpret the protective effect of ANC utilisation on childhood ARIs within the broader environmental and socio-economic context of many LMICs. Widespread exposure to biomass fuel smoke, poor ventilation, malnutrition, and crowded living conditions represents a cumulative risk environment that may attenuate or mask the modest benefits associated with ANC utilisation. These pervasive determinants can overwhelm preventive gains achieved through health education or maternal immunisation delivered during ANC visits. Consequently, improving child respiratory outcomes will likely require integrated strategies that strengthen ANC while simultaneously addressing these risk factors, such as household air quality, nutrition, and access to postnatal and community-based child health services. Multisectoral approaches that combine health-system interventions with environmental and social measures could therefore provide the most sustainable reductions in childhood ARI burden.

Notably, two studies focused on pertussis in infants and found no significant association between maternal ANC attendance and infant pertussis [30,38]. This is understandable, as the prevention of pertussis in young infants depends primarily on the timely administration of maternal Tdap vaccination rather than ANC attendance alone. The Tdap vaccine must be given during late pregnancy (ideally between 27 and 36 weeks of gestation) to maximise antibody transfer to the newborn [48]. In the study by Winter et al. [38] only 43.5% of women in the US cohort received the Tdap vaccine within this optimal window; the remaining were immunised too early, too late, or postpartum, significantly reducing the protective benefit for their infants. These findings indicate that for pertussis prevention, the specific content and timing of antenatal care (ANC) interventions, such as appropriate vaccinations, are more critical than ANC attendance alone. ANC attendance alone may not reduce infant pertussis risk unless it effectively facilitates timely immunisation, highlighting that the quality and content of ANC, particularly with regard to immunisation counselling and delivery, are more critical than attendance alone.

Additionally, in nearly all included studies, residual confounding or inadequate adjustment for confounders may have contributed to inconsistent findings. Although several included studies were published after the onset of the COVID-19 pandemic, none explicitly assessed pandemic-related disruptions to antenatal or child health services. While no systematic differences were observed between pre- and post-pandemic studies, it is plausible that the pandemic influenced maternal care-seeking behaviours and healthcare accessibility in many settings. Future research should examine how such service disruptions and behavioural changes may have affected antenatal care utilisation and subsequent child respiratory outcomes.

This systematic review and meta-analysis address a critical gap in the literature as it is the first to synthesise evidence in this area. Employing both narrative synthesis and meta-analysis, it provides a comprehensive and methodologically rigorous summary of the available data, guided by SWiM principles. Due to limited data and heterogeneity in how ANC exposure was defined across different outcome groups, narrative synthesis was used to integrate findings from diverse study designs, methodologies, and contexts. In addition, a meta-analysis was performed for pneumonia outcomes, providing a pooled estimate of effect sizes to complement the qualitative findings.

Despite its strengths, this review has several acknowledged limitations. The evidence base was relatively small and comprised only observational studies. This limits clear causal inference between ANC and ARIs. In most of the included studies, data collection, particularly for both ANC exposure and ARI outcomes, relies on maternal recall, which may introduce recall bias and reporting bias. Additionally, there was inconsistency and a lack of clarity in how ARI cases were defined across studies; for instance, while some studies explicitly described how they assessed ARI, others used varying and poorly defined assessment criteria. Furthermore, our focus was on studies employing any regression techniques, though we acknowledged several limitations, particularly regarding sample size and lack of adjustments for confounding variables. We could not perform meta-analysis for pertussis and ARI outcomes due to inconsistency in categorisations of exposure (ANC) and variations in case definitions of outcomes across studies, which limits our ability to estimate an overall pooled effect. Our review was limited to articles published in English, which could introduce language bias, and may have missed potential articles published in other languages. These limitations suggest that findings should be interpreted with appropriate caution. Future studies should incorporate all those methodological limitations to improve the quality of evidence.

## 5. Conclusions

We emphasise the crucial role of ANC utilisation in supporting maternal and child health, particularly in low-resource settings where access to preventive services remains limited. The findings suggest that regular ANC contact provides an essential platform for delivering interventions that can reduce the risk of childhood pneumonia and other ARIs; however, the evidence remains limited and inconsistent regarding pertussis. Importantly, ANC attendance alone is insufficient; its protective potential depends on the delivery of high-quality, evidence-based services such as maternal vaccination, nutritional supplementation, infection screening, and health education. Strengthening ANC uptake, together with improvements in service quality, continuity of care, and postnatal follow-up, may therefore enhance the effectiveness of these preventive interventions. Future research should investigate how these specific components contribute to ARI prevention and explore differences across diverse populations, including urban and rural settings, while accounting for social, cultural, and behavioural factors that influence care-seeking. Such efforts will enhance the effectiveness and equity of ANC programs, contributing to a reduction in under-five morbidity and mortality.

## Figures and Tables

**Table 1:** Characteristics of included studies.

Author, Year of Publication	Continent/Country	Country’s Income Level	Population	Sample Size	Study Design	Objective of the Study	Exposure	Measurement of Outcome	QAS
Ahmed et al., 2024 [37]	Africa/25 SSA countries	LMIC	Under five years	253,167	Cross-sectional	To assess the modifiable risk factors of ARI and diarrhoea using DHS data from 25 SSA countries	ANC	ARI was measured based on cough, along with rapid and shallow breathing	Moderate risk
Yadate et al., 2023 [33]	Africa/Ethiopia	LMIC	2 to 59 months	398	Case–control	To examine the determinants of pneumonia using hospital records	ANC	Pneumonia was measured based on cough, grunting, difficulty breathing, and age-specific fast breathing or consolidation/inflation using IMNCI guidelines	Moderate risk
Bokoro et al., 2022 [31]	Africa/Ethiopia	LMIC	Under five years	9917	Cross-sectional	To assess the risk factors of ARI and diarrhoea using DHS data	ANC	The ARI measurement method was not stated.	Moderate risk
Buchanan et al., 2020 [39]	Europe/England	HIC	Under two years	1505	Cross-section	To identify risk factors for respiratory tract infection (RTI) attendance and primary care consultation using Building Blocks data	ANC	The RTI measurement method was not stated	Moderate risk
Turkson & Ahiabor, 2020 [35]	Africa/Ghana	LMIC	Under five years	3057	Cross-sectional	To assess the impact of natal care and maternity leave on ARI using DHS data	Perinatal care	The ARI measurement method was not stated	Low risk
Winter & Harriman, 2018 [30]	North America/US	HIC	Mother-Under four infant pairs	994,244	Case–control	To examine risk factors for pertussis using pertussis surveillance data linked to birth certificate records	Perinatal care	Pertussis was measured based on acute cough illness or laboratory detection of *Bordetella pertussis*	Moderate risk
Yang et al., 2018 [34]	Asia/China	LMIC	Neonates	953	Cross-sectional	To assess the prevalence and risk factors for pneumonia using hospital records	ANC	Pneumonia was measured using bacterial detection via blood culture	High risk
Workineh et al., 2017 [32]	Africa/Ethiopia	LMIC	Under two years	558	Case–control	To assess determinants of pneumonia using health centre records during the data collection period	ANC	Pneumonia was measured using IMNCI guidelines, but specific diagnostic criteria were not stated	High risk
							Time of ANC booking		
Winter et al., 2016 [38]	North America/US	HIC	Mother-infant pairs	74,504	Cross-sectional	To assess the effectiveness of prenatal and postnatal TdaP vaccination on pertussis using surveillance data	Perinatal care	Pertussis was measured based on an acute respiratory illness or laboratory detection of *Bordetella pertussis*	Moderate risk
Johnson et al., 2010 [36]	Africa/Rwanda	LMIC	Under five years	3745	Cross-sectional	To assess the determinants of poor health outcomes based on OVC status using DHS data	Perinatal care	ARI was measured based on cough accompanied by short and rapid breathing	Moderate risk
Choudhury et al., 201 [29]	Asia/Bangladesh	LMIC	Neonates (<28 days)	100	Case–control	To assess the determinants of pneumonia using hospital records	ANC	Pneumonia was measured based on rapid breathing (>60 breaths/minute), severe chest indrawing, and chest X-ray showing consolidation or patchy opacity	High risk

Key: ARI = acute respiratory infection, HIC = high-income country, IMNCI = Integrative Management of Newborn and Childhood Illness, IQR = Interquartile Range, LMIC = low- and middle-income country, QAS = quality assessment score, SD = Standard Deviation, Tdap = Tetanus, diphtheria and acellular pertussis, US = United States.

**Table 2:** Findings of included studies.

Author, Publication Year	Outcomes	% of Outcome or Case/Control Ratio	Exposure Definition (% or Mean)	Effect Estimate (β, OR 95% CI)		*p*-Value	Confounder Adjustment
Ahmed et al., 2024 [37]	ARI		≥4 ANC visits (56.8)	≥4 ANC visits	Ref		Perceived birth weight, breastfeeding, maternal education, employment, place of birth, household wealth index, type of toilet, cooking fuel, and residence
		4.6	≤3 ANC visits (43.2)	≤3 ANC visits	aOR = 0.96 (0.90, 1.02)	Not reported	
Yadate et al., 2023 [33]	Pneumonia	1:1	No (9.8)	No	Ref.		Mother’s age, residence, awareness of domestic smoking, children’s ages and birth weights, breastfeeding, zinc and vitamin supplementation, history of diarrhea and URIs, latrine usage, proper handwashing practices, source of light, cooking fuel source, cooking location, availability and number of open windows, and number of people sleeping in the same room
			Yes (90.2)	Yes	cOR = 1.43 (0.84, 2.24)	<0.2	
Bokoro et al., 2022 [31]			≥5 ANC visits (20.9)	≥5 ANC visits	Ref		Child size, residence, mothers’ education, wealth index, vaccination status
	ARI	16.0	<5 ANC visits (45.3)	<5 ANC visits	aOR = 1.20	0.207	
			No ANC visits (33.7)	No ANC visit	aOR = 1.15	0.169	
Buchanan et al., 2020 [39]	RTI (A & E)	20.8		Number of ANC visits	aOR = 0.96 (0.92, 0.99)	Not reported	Mother’s alcohol consumption, season at birth, neonatal unit admission, index of multiple deprivation quintile, adaptive function
	RTI (PHC)	77.5	Number of ANC visits (*)		cOR = 1.02 (0.98, 1.06)		
	RTI (HA)	8.6			cOR = 1.00 (0.96, 1.04)		
Turkson & Ahiabor, 2020 [35]	ARI	24.3	Number of ANC visits (mean (SD) = 6.8 (6.53))	Number of ANC visits	Β = −0.0021, marginal effect = −0.006	0.547	Postnatal care, place of delivery, child death history, birth experience, wealth index, maternity leave, residence, marital status, child age, age of mother, maternal education, vaccination
Winter & Harriman, 2018 [30]	Pertussis		0–5 ANC visits (4.00	0–5 ANC visits	cOR = 1.3 (0.9, 1.8)	0.616	Sex of baby, term at birth, birth weight, race of mother, mother born outside of the US, birth order, payer, age of mother
		1:1316	6–12 ANC visits (60.0)	6–12 ANC visits	cOR = 0.9 (0.8, 1.1)		
			13–18 ANC visits (31.5)	13–18 ANC visits	cOR = 1.0 (0.9, 1.2)		
			≥19 ANC visits (4.5)	≥19 ANC visits	cOR = 1.1 (0.5, 1.5)		
Yang et al., 2018 [34]			Adequate ANC (70.0)	Adequate ANC	Ref		Place of delivery, fever at birth, gynaecological problem during pregnancy, duration of labour, mode of delivery, rupture of membrane, type of person-assisted delivery, birth weight, neonatal resuscitation, foul-smelling liquor
	Pneumonia	14.0	Inadequate ANC (30.0)	Inadequate ANC	aOR = 24.90 (21.20, 28.60)	<0.001	
Workineh et al., 2017 [32]	Pneumonia		No (10.8)	No	Ref		Marital and educational status of the mother, children without young siblings, child vaccination, time of breastfeeding initiation, vitamin A utilisation, status of foetus during delivery, ever breastfeeding, types of breastfeeding, and time of breastfeeding
		1:2	Yes (89.2)	Yes	aOR = 1.97 (0.34, 11.40)	Not reported	
			Within 4 months (70.1)	Within 4 months	Ref		
			After 4 months (29.9)	After 4 months	aOR = 0.67 (0.41, 1.07)		
Winter et al., 2016 [38]	Pertussis (< 8 weeks of age)	0.03	Number of ANC visits (mean (IQR) = 12.4 (10–14) in prenatal Tdap and 11.8 (10–14) in postpartum Tdap)	Number of ANC visits	aOR = 1.05 (0.97, 1.13)	Not reported	Ethnicity, time of mother’s pertussis vaccination, payer, child sex, birth weight, term of birth, and age of mother.
	Pertussis (≤12 weeks of age)	0.05		Number of ANC visits	aOR = 1.02 (0.97, 1.10)		
Johnson et al., 2010 [36]	ARI	17.3	Unskilled care (% not reported)	Unskilled	Ref		Orphaned and vulnerable status, fever, cough, and diarrhea, breastfeeding, source of water, age and sex of baby, birth interval, household structure, number of household members, maternal education, region, and residence
			Skilled care (% not reported)	Skilled care	aOR = 0.78 (0.61, 0.99)	Not reported	
Choudhury et al., 2010 [29]	Pneumonia	1:1	Adequate/>3 ANC visits (45.0)	Adequate	Ref		Place of delivery, intrapartum fever, resuscitation required, obstetric problem of the mother, prolonged labour
			Inadequate/<3 ANC visits (55.0)	Inadequate	aOR = 168.9 (8.0, 3559.2)	0.001	

Key: A&E = Accident and Emergency, β = Coefficient, aOR = Adjusted Odds Ratio, cOR = Crude Odds Ratio, HA = Hospital Admission, PHC = Primary Healthcare Consultation, Ref = Reference, RTI = Respiratory Tract Infection, URIs = upper respiratory infections. * Among Primary health care participants with RTI, the mean number of ANC visits was 10.23 (SD = 3.85), while those without RTI had a mean of 10.50 (SD = 3.75). Among accident and emergency participants with RTI, the mean was 10.42 (SD 3.59); among those without RTI, it was 9.97 (SD = 4.36). For hospital admission participants, the mean ANC visits were 10.37 (SD 3.74), compared to 10.02 (SD = 3.67) among those without RTI.

## Data Availability

Not applicable.

## Supplementary Materials

The following supporting information can be downloaded at https://www.mdpi.com/article/10.3390/ijerph22111627/s1. Table S1: PRISMA 2020 checklist; Table S2: Preliminary search strategy from Medline; Table S3: Quality assessment score of included studies; Table S4: Excluded articles after full text review; Table S5: Key Variables and evidence requiring standardisation for future research.

## Abbreviations

ARI	Acute Respiratory Infections
ANC	Antenatal Care
CI	Confidence Interval
DHS	Demographic and Health Survey
LMICs	Low- and Middle-Income Countries
NOS	Newcastle-Ottawa Scale
PRSMA	Preferred Reporting Items for Systematic Reviews and Meta-Analyses (PRISMA)
SWiM	Synthesis Without Meta-analysis
