# Supplementary material for: The Effect of Maternal Antenatal Care Utilisation on Childhood Acute Respiratory Infection: A Systematic Review and Meta-Analysis
Source: Int J Environ Res Public Health. 2025 Oct 26;22(11):1627. doi: 10.3390/ijerph22111627 (PMC12652360; doi:10.3390/ijerph22111627)
Supplement: Supplementary file 1 [file ijerph-22-01627-s001.zip › ijerph-3830150-supplementary.pdf]

Supplementary materials

The Effect of Maternal Antenatal Care Utilisation on Childhood Acute Respiratory Infection: A Systematic Review and Meta-analysis

Table S1: PRISMA 2020 checklist

Section and Topic	Item #	Checklist item	Location where item is reported
**TITLE**			
Title	1	Identify the report as a systematic review.	1
**ABSTRACT**			
Abstract	2	See the PRISMA 2020 for Abstracts checklist.	1
**INTRODUCTION**			
Rationale	3	Describe the rationale for the review in the context of existing knowledge.	2 to 3
Objectives	4	Provide an explicit statement of the objective(s) or question(s) the review addresses.	3
**METHODS**			
Eligibility criteria	5	Specify the inclusion and exclusion criteria for the review and how studies were grouped for the syntheses.	3
Information sources	6	Specify all databases, registers, websites, organisations, reference lists and other sources searched or consulted to identify studies. Specify the date when each source was last searched or consulted.	4
Search strategy	7	Present the full search strategies for all databases, registers and websites, including any filters and limits used.	4
Selection process	8	Specify the methods used to decide whether a study met the inclusion criteria of the review, including how many reviewers screened each record and each report retrieved, whether they worked independently, and if applicable, details of automation tools used in the process.	4
Data collection process	9	Specify the methods used to collect data from reports, including how many reviewers collected data from each report, whether they worked independently, any processes for obtaining or confirming data from study investigators, and if applicable, details of automation tools used in the process.	4
Data items	10a	List and define all outcomes for which data were sought. Specify whether all results that were compatible with each outcome domain in each study were sought (e.g. for all measures, time points, analyses), and if not, the methods used to decide which results to collect.	4
	10b	List and define all other variables for which data were sought (e.g. participant and intervention characteristics, funding sources). Describe any assumptions made about any missing or unclear information.	4
Study risk of bias assessment	11	Specify the methods used to assess risk of bias in the included studies, including details of the tool(s) used, how many reviewers assessed each study and whether they worked independently, and if applicable, details of automation tools used in the process.	4

Section and Topic	Item #	Checklist item	Location where item is reported
Effect measures	12	Specify for each outcome the effect measure(s) (e.g. risk ratio, mean difference) used in the synthesis or presentation of results.	5
Synthesis methods	13a	Describe the processes used to decide which studies were eligible for each synthesis (e.g. tabulating the study intervention characteristics and comparing against the planned groups for each synthesis (item #5)).	5
	13b	Describe any methods required to prepare the data for presentation or synthesis, such as handling of missing summary statistics, or data conversions.	5
	13c	Describe any methods used to tabulate or visually display results of individual studies and syntheses.	5
	13d	Describe any methods used to synthesize results and provide a rationale for the choice(s). If meta-analysis was performed, describe the model(s), method(s) to identify the presence and extent of statistical heterogeneity, and software package(s) used.	5
	13e	Describe any methods used to explore possible causes of heterogeneity among study results (e.g. subgroup analysis, meta-regression).	5
	13f	Describe any sensitivity analyses conducted to assess robustness of the synthesized results.	-
Reporting bias assessment	14	Describe any methods used to assess risk of bias due to missing results in a synthesis (arising from reporting biases).	-
Certainty assessment	15	Describe any methods used to assess certainty (or confidence) in the body of evidence for an outcome.	-
**RESULTS**			
Study selection	16a	Describe the results of the search and selection process, from the number of records identified in the search to the number of studies included in the review, ideally using a flow diagram.	5 and Figure 1
	16b	Cite studies that might appear to meet the inclusion criteria, but which were excluded, and explain why they were excluded.	Supplementary material
Study characteristics	17	Cite each included study and present its characteristics.	Table 1
Risk of bias in studies	18	Present assessments of risk of bias for each included study.	Supplementary material
Results of individual studies	19	For all outcomes, present, for each study: (a) summary statistics for each group (where appropriate) and (b) an effect estimate and its precision (e.g. confidence/credible interval), ideally using structured tables or plots.	Table 2
Results of syntheses	20a	For each synthesis, briefly summarise the characteristics and risk of bias among contributing studies.	10 to 15
	20b	Present results of all statistical syntheses conducted. If meta-analysis was done, present for each the summary estimate	13

Section and Topic	Item #	Checklist item	Location where item is reported
		and its precision (e.g. confidence/credible interval) and measures of statistical heterogeneity. If comparing groups, describe the direction of the effect.	
	20c	Present results of all investigations of possible causes of heterogeneity among study results.	13
	20d	Present results of all sensitivity analyses conducted to assess the robustness of the synthesized results.	-
Reporting biases	21	Present assessments of risk of bias due to missing results (arising from reporting biases) for each synthesis assessed.	-
Certainty of evidence	22	Present assessments of certainty (or confidence) in the body of evidence for each outcome assessed.	-
**DISCUSSION**			
Discussion	23a	Provide a general interpretation of the results in the context of other evidence.	15 to 17
	23b	Discuss any limitations of the evidence included in the review.	18
	23c	Discuss any limitations of the review processes used.	18
	23d	Discuss implications of the results for practice, policy, and future research.	18
**OTHER INFORMATION**			
Registration and protocol	24a	Provide registration information for the review, including register name and registration number, or state that the review was not registered.	Not registered
	24b	Indicate where the review protocol can be accessed, or state that a protocol was not prepared.	N/A
	24c	Describe and explain any amendments to information provided at registration or in the protocol.	N/A
Support	25	Describe sources of financial or non-financial support for the review, and the role of the funders or sponsors in the review.	19
Competing interests	26	Declare any competing interests of review authors.	19
Availability of data, code and other materials	27	Report which of the following are publicly available and where they can be found: template data collection forms; data extracted from included studies; data used for all analyses; analytic code; any other materials used in the review.	19

From: Page MJ, McKenzie JE, Bossuyt PM, Boutron I, Hoffmann TC, Mulrow CD, et al. The PRISMA 2020 statement: an updated guideline for reporting systematic reviews. BMJ 2021;372:n71. doi: 10.1136/bmj.n71. This work is licensed under CC BY 4.0. To view a copy of this license, visit https://creativecommons.org/licenses/by/4.0/

Table S2: Preliminary search strategy from Medline

S.no	Concept	**Medline**
1	Population	Infant, newborn/ OR Infant/ OR pediatrics/ OR (pediatric* OR "children under 5" OR "under-five" OR "infants" OR "toddlers" OR "newborns" OR "neonates" OR "young children" OR "preschool children" OR "early childhood" OR "children aged 0-5 years")
2	Exposure	Prenatal Care/ OR perinatal care/ OR Maternal Health Services/ OR ("Prenatal Care" OR "Pre natal care" OR "Antenatal care" OR "Ante natal care" OR "Perinatal Care" OR "Peri natal Care" OR "pregnanc* care" OR "Ante partum care" OR "Maternal Health Service" OR "Maternal Health Services" OR "Maternal healthcare*" Or "Maternal health behavi?r" OR "maternal health seeking behavi?r").mp
3	Outcomes	Bronchitis/ OR Laryngitis/ OR Pneumonia/ OR Common cold/ OR Rhinitis/ OR Sinusitis/ OR Tonsillitis/ OR Epiglottitis/ OR Pharyngitis/ or Otitis/ OR Whooping Cough/ OR ("respiratory tract infection*" OR "Respiratory Infection*" OR "Respiratory System Infection*" OR "upper respiratory tract infection*" OR "lower respiratory tract infection*" OR bronchitis OR "common cold" OR laryngitis OR pharyngitis OR epiglottitis OR pneumonia OR rhinitis OR sinusitis OR tonsillitis OR otitis OR Tracheitis OR "whooping cough")
Final	Combination	1 AND 2 AND 3

Table S3: quality assessment score of included studies

Quality assessment score for cross-sectional studies								
Criteria	First author /publication year	Ahmed, 2024	Bokoro, 2022	Buchanan, 2020	Turkson, 2020	Yang, 2018	Winter, 2016	Johnson, 2010
Selection	Representativeness of the sample	*	*	*	*	*	*	*
	Is the sample size adequate?	*	*	*	*	*	*	*
	Non-respondents			*			*	
	Ascertainment of the exposure (risk factor)	*	*	*	*		*	*
Comparability	The study controls for the most important factor (Residence, maternal age, and maternal educational status)				*			
	The study control for any additional factor				*			
Outcome	Assessment of outcome	*				*	*	*
	Statistical test	*	*		*	*		*
Total score		5	4	4	6	4	5	5

Quality assessment score for case-control studies					
**Criteria**	First author/ publication year	Choudhury, 2010	Workineh, 2016	Winter, 2018	Yadate, 2023
Selection	Is the case definition adequate?	*		*	*
	Representativeness of the cases		*	*	*
	Selection of control	*	*	*	
	Definition of control	*			*
Comparability	The study controls for the most important factors (Residence, maternal age, and maternal educational status)				
	The study control for any additional factor				
Exposure	Exposures were measured using standard tools, medical records, or structural tools				
	Same method of ascertainment for cases and controls	*	*		
	Non-response rate		*	*	*
Total score		4	4	4	4

NEWCASTLE - OTTAWA QUALITY ASSESSMENT SCALE CROSS-SECTIONAL STUDIES

Selection (Maximum 5 * stars)

1. Representativeness of the sample
   A. Truly representative of the average target population (all subjects or random sampling) *
   B. Somewhat representative of the average target population (non-random sampling) *
   C. Selected group of users
   D. No description of the sampling strategy
2. Sample size
   A. Justified and satisfactory *
   B. Not justified
3. Non-respondents
   A. Comparability between respondents and non-respondents characteristics is established, and the response rate is satisfactory *
   B. The response rate is unsatisfactory, or the comparability between respondents and nonrespondents is unsatisfactory
   C. No description of the response rate or the characteristics of the responders and the nonresponders
4. Ascertainment of the exposure (risk factor)
   A. Validated measurement tool **
   B. Non-validated measurement tool, but the tool is available or described *
   C. No description of the measurement tool

Comparability (Maximum 2 * stars)

1. The subjects in different outcome groups are comparable, based on the study design or analysis. Confounding factors are controlled.
   A. The study controls for the (Residence, maternal age, maternal educational status) *
   B. The study control for any additional factor *

Outcome (Maximum 3 * stars)

1. Assessment of the outcome
   A. Independent blind assessment **
   B. Record linkage **
   C. Self-report *
   D. No description
2. Statistical test
   A. The statistical test used to analyze the data is clearly described and appropriate, and the measurement of the association is presented, including confidence intervals and the probability level (p-value) *
   B. The statistical test is not appropriate, not described, or incomplete.

NEWCASTLE - OTTAWA QUALITY ASSESSMENT SCALE CASE-CONTROL STUDIES

Selection (Maximum 4 * stars)

1. Is the case definition adequate?
   A. yes, with independent validation *
   B. yes, eg. record linkage or based on self-reports
   C. no description
2. Representativeness of the cases
   A. consecutive or obviously representative series of cases *
   B. potential for selection biases or not stated
3. Selection of Controls
   A. community controls *
   B. hospital controls
   C. no description
4. Definition of Controls
   A. no history of disease (endpoint) *
   B. no description of the source

Comparability (Maximum 2 * stars)

1. Comparability of cases and controls on the basis of the design or analysis
    - A. study controls for (Residence, maternal age, maternal educational status) *
    - B. study controls for any additional factor *

Exposure (Maximum 3 * stars)

1. Ascertainment of exposure
    - A. secure record (eg surgical records) *
    - B. structured interview where blind to case/control status *
    - C. interview not blinded to case/control status.
    - D. written self-report or medical record only.
    - E. no description
2. Same method of ascertainment for cases and controls
    - A. yes *
    - B. no
    - C. not mentioned
3. Non-response rate
    - A. same rate for both groups *
    - B. non-respondents described.
    - C. rate different and no designation

Table S4: Excluded articles after full text review

S.No	Studies	Reason for exclusion
1	Kaali S, Jack DW, Mujtaba MN, Chillrud SN, Kinney PL, Kaali EB, et al. Identifying sensitive windows of prenatal household air pollution on birth weight and infant pneumonia risk to inform future interventions. Environment International. 2023 Aug 1;178:108062.	Didn't report the exposure variable of interest/ not reported ANC use
2	Loddo F, Nauleau S, Lapalus D, Tardieu S, Bernard O, Boubred F. Association of maternal gestational vitamin D supplementation with respiratory health of young children. Nutrients. 2023 May 19;15(10):2380.	The respiratory health outcome was not limited to ARI, but also other non-infectious respiratory conditions
3	Foo D, Sarna M, Pereira G, Moore HC, Regan AK. Longitudinal, population-based cohort study of prenatal influenza vaccination and influenza infection in childhood. Vaccine. 2022 Jan 28;40(4):656-65.	Didn't report the exposure variable of interest/not reported ANC use
4	Zar HJ, MacGinty R, Workman L, Burd T, Smith G, Myer L, et al. Klebsiella pneumoniae lower respiratory tract infection in a South African birth cohort: a longitudinal study. International Journal of Infectious Diseases. 2022 Aug 1;121:31-8.	Didn't report the exposure variable of interest/not reported ANC use
5	Kinney PL, Asante KP, Lee AG, Burkart K, Boamah-Kaali E, et al. Prenatal and postnatal household air pollution exposures and pneumonia risk: evidence from the Ghana Randomized Air Pollution and Health Study. Chest. 2021 Nov 1;160(5):1634-44.	Didn't report exposure variable of interest/not reported ANC use
6	Rowland R, Sass Z, Ponsonby AL, Pezic A, Tang ML, Vuillermin P, et al. Burden of infection in Australian infants. Journal of Paediatrics and Child Health. 2021 Feb;57(2):204-11.	Didn't report the exposure variable of interest/not reported ANC use
7	Le Roux DM, Nicol MP, Myer L, Vanker A, Stadler JA, von Delft E, et al. Lower respiratory tract infections in children in a well-vaccinated South African birth cohort: spectrum of disease and risk factors. Clinical Infectious Diseases. 2019 Oct 15;69(9):1588-96.	Didn't report the exposure variable of interest/not reported ANC use
8	Alfonso VH, Bandoli G, von Ehrenstein O, Ritz B. Early folic acid supplement initiation and risk of adverse early childhood respiratory health: a population-based study. Maternal and Child Health Journal. 2018 Jan;22:111-9.	The exposure variable was not included in the regression analysis. In addition, no descriptive statistics that demonstrated the relationship between the exposure and outcome

9	Soh SE, Goh A, Teoh OH, Godfrey KM, Gluckman PD, Shek LP, et al. Pregnancy trimester-specific exposure to ambient air pollution and child respiratory health outcomes in the first 2 years of life: effect modification by maternal pre-pregnancy BMI. International journal of environmental research and public health. 2018 May;15(5):996.	Didn't report the exposure variable of interest/not reported ANC use
10	Pina JC, Moraes SA, Freitas IC, Mello DF. Role of Primary Health Care in child hospitalization due to pneumonia: a case-control study. Revista latino-americana de enfermagem. 2017;25:e2892.	Didn't report the exposure variable of interest/not reported ANC use
11	Winter K, Cherry JD, Harriman K. Effectiveness of prenatal tetanus, diphtheria, and acellular pertussis vaccination on pertussis severity in infants. Clinical Infectious Diseases. 2016 Sep 13:ciw633.	The exposure variable was not included in the regression analysis. In addition, no descriptive statistics that demonstrated the relationship between the exposure and outcome
12	Chien LN, Chiou HY, Wang CW, Yeh TF, Chen CM. Oligohydramnios increases the risk of respiratory hospitalization in childhood: a population-based study. Pediatric research. 2014 Apr;75(4):576-81.	Didn't report an exposure variable of interest. Additionally, respiratory hospitalisation does not explicitly specify whether it is caused by an acute respiratory infection (ARI) or other non-infectious respiratory conditions.
13	Belderbos ME, Houben ML, Wilbrink B, Lentjes E, Bloemen EM, Kimpen JL, et al. Cord blood vitamin D deficiency is associated with respiratory syncytial virus bronchiolitis. Pediatrics. 2011 Jun 1;127(6):e1513-20.	Didn't report the exposure variable of interest/ not reported ANC use
14	Prietsch SO, Fischer GB, César JA, Lempek BS, Barbosa Jr LV, Zogbi L, et al. Acute lower respiratory illness in under-five children in Rio Grande, Rio Grande do Sul State, Brazil: prevalence and risk factors. Cadernos de saude publica. 2008;24:1429-38.	Didn't report the exposure variable of interest/not reported ANC use
15	Mahon BE, Ehrenstein V, Nørgaard M, Pedersen L, Rothman KJ, Sørensen HT. Perinatal risk factors for hospitalization for pneumococcal disease in childhood: a population-based cohort study. Pediatrics. 2007 Apr 1;119(4):e804-12.	Didn't report the exposure variable of interest/not reported ANC use
16	Coles CL, Fraser D, Givon-Lavi N, Greenberg D, Gorodischer R, Bar-Ziv J, et al. Nutritional status and diarrheal illness as independent risk factors for alveolar pneumonia. American journal of epidemiology. 2005 Nov 15;162(10):999-1007.	Didn't report the exposure variable of interest/not reported ANC use
17	Khadivzadeh T, Parsai S. Effect of exclusive breastfeeding and complementary feeding on infant growth and morbidity. EMHJ-Eastern Mediterranean Health Journal, 10 (3), 289-294, 2004. 2004.	Didn't report the exposure variable of interest/not reported ANC use

Key Variables and Evidence Requiring Standardisation for Future Research

This table highlights inconsistencies in how ANC exposure and childhood respiratory outcomes were defined and reported in the included studies. It also offers recommendations for standardising these definitions to enhance comparability and data quality in future research (Table S5).

Table S5: Key Variables and evidence requiring standardisation for future research.

Domain	Variable/Indicator	Current Variation Across Studies	Recommendation for Standardisation
**ANC Exposure**	Number of ANC visits	Ranged from 0–19+ visits; some used binary (yes/no)	Align with WHO's ANC models, report both numbers and timing.
	Timing of first visit	<4 months vs ≥4 months; inconsistently reported	Standardise trimester-based classification
	ANC provider type	Skilled vs unskilled; unclear in some studies	Use the WHO skilled-attendant definition
**Outcomes (ARI/Pneumonia)**	Case definition	Varied: IMNCI criteria, self-report, clinical diagnosis, lab-confirmed	Adopt the WHO ARI/pneumonia diagnostic criteria

WHO=World Health Organisation, ANC=antenatal care
